# A de novo 2.9 Mb interstitial deletion at 13q12.11 in a child with developmental delay accompanied by mild dysmorphic characteristics

**DOI:** 10.1186/s13039-014-0092-5

**Published:** 2014-12-03

**Authors:** Magdalini Lagou, Ioannis Papoulidis, Sandro Orru, Vasileios Papadopoulos, George Daskalakis, Maria Kontodiou, Eleftherios Anastasakis, Michael B Petersen, George Kitsos, Loretta Thomaidis, Emmanouil Manolakos

**Affiliations:** Bioiatriki S.A., Laboratory of Genetics, Athens, Greece; Eurogenetica S.A., Laboratory of Genetics, Michalakopoulou 125& Vervainon 14, 11527 Athens, Thessaloniki Greece; Department of Medical Genetics, University of Cagliari, Binaghi Hospital, Cagliari, Italy; Department of Obstetrics & Gynecology, University of Patra, Patra, Greece; 1st Department of Obstetrics & Gynecology, University of Athens, Athens, Greece; Department of Gynecology, Naval Hospital of Athens, Athens, Greece; Department of Clinical Medicine, The Faculty of Medicine, Aalborg University Hospital, Aalborg, Denmark; Department of Ophthalmology, University of Ioannina, Ioannina, Greece; Developmental Assessment Unit, 2nd Department of Pediatrics, P. & A. Kyriakou Children’s Hospital, National and Kapodistrian University of Athens, School of Medicine, Athens, Greece

**Keywords:** 13q12.11, Array-CGH, Deletion, Mild dysmorphic features

## Abstract

**Background:**

Proximal deletions in the 13q12.11 region are very rare. Much larger deletions including this region have been described and are associated with complex phenotypes of mental retardation, developmental delay and various others anomalies.

**Results:**

We report on a 3-year-old girl with a rare 2.9 Mb interstitial deletion at 13q12.11 due to a de novo unbalanced t(13;14) translocation. She had mild mental retardation and relatively mild dysmorphic features such as microcephaly, flat nasal bridge, moderate micrognathia and clinodactyly of 5^th^ finger. Molecular karyotyping revealed a deletion on the long arm of chromosome 13 as involving sub-bands 13q12.11, a deletion of about 2.9 Mb.

**Discussion:**

The clinical application of array-CGH has made it possible to detect submicroscopical genomic rearrangements that are associated with varying phenotypes.The description of more patients with deletions of the 13q12.11 region will allow a more precise genotype-phenotype correlation.

## Background

Genetic testing, such as chromosome analysis, is an important diagnostic tool for patients with unexplained developmental delay, dysmorphisms and other developmental disabilities. The development of novel high-resolution molecular methods such as array based comparative genomic hybridization (array-CGH) increased the resolution of chromosomal studies enabling the detection of submicroscopic chromosome aberrations, otherwise not detectable by conventional cytogenetic techniques [1].

Herein, we report the case of a 5 ½ years old female child with developmental delay and a relatively mild phenotype. The karyotype revealed a *de novo* translocation between chromosomes 13 and 14. Further analysis using array-CGH, however, revealed a 2.9 MB deletion within the 13q12.11 region. To date, two similar cases have been reported with an interstitial deletion within the 13q12.11 region presenting similar phenotypic characteristics and overall development and intellect [2,3].

## Case presentation

### Case report

The patient was a female child born to unrelated healthy young parents after an uncomplicated 36 weeks pregnancy. She was referred to our unit for developmental assessment at the age of 3 years and 2 months because of speech and language delay.

**Figure 1:**
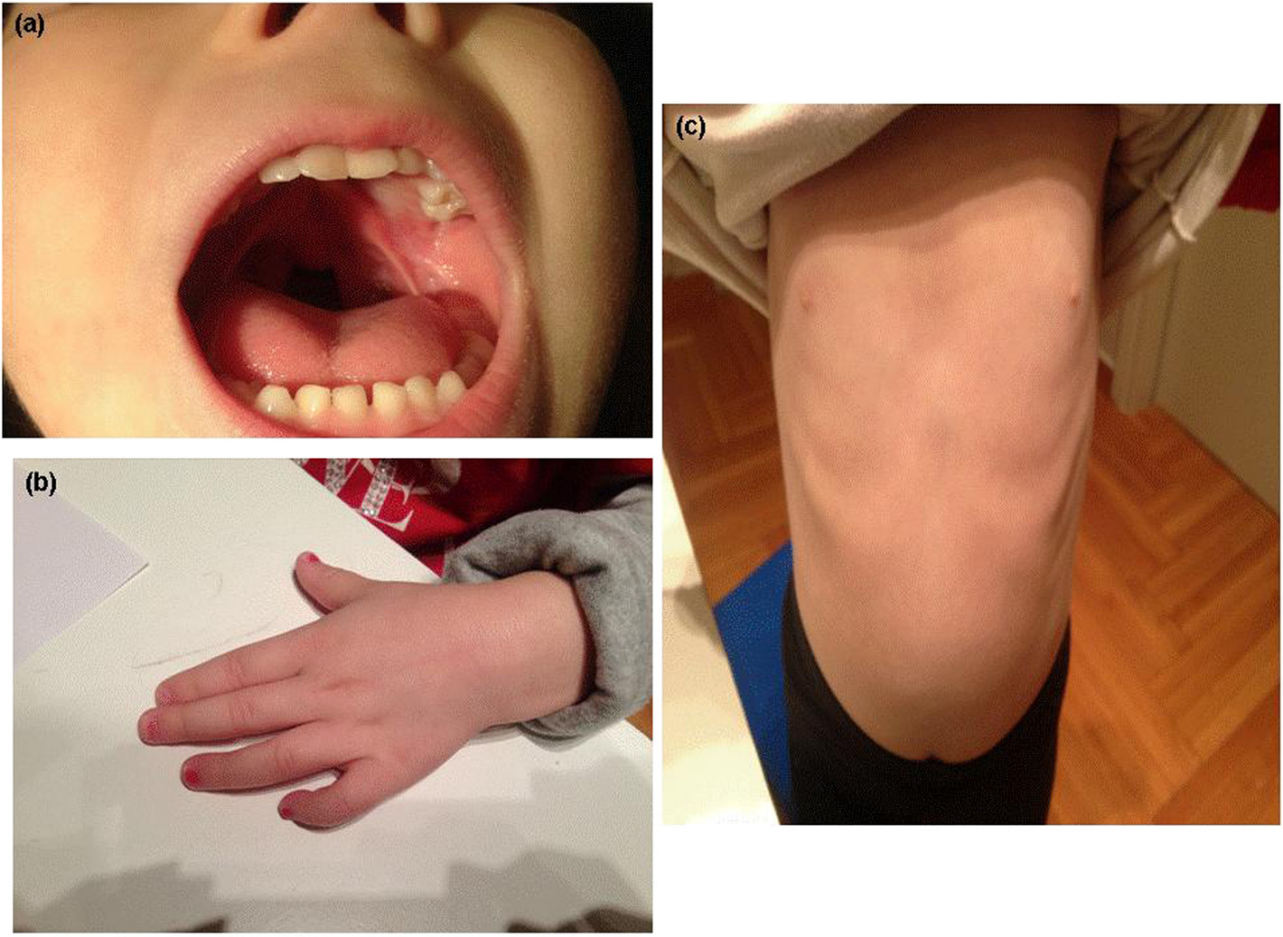
**Views of the patient at the age of 5 years and 5 months. (a)** Frontal view of the face of the patient. Incomplete soft cleft palate surgically corrected at the age of 14 months. **(b)** Dorsal view of the hand of the patient showing clinodactyly of 5^th^ finger. **(c)** Frontal view of the patient showing the widely spaced nipples.

Delivery was by cesarean section because of premature rupture of membranes. Birth weight was 3,150 kg (50^th^ percentile**)**, length was 47 cm (10^th^ percentile**)** and head circumference (H.C.) was 32 cm (3^rd^ percentile). At birth she presented with incomplete soft cleft palate which was surgically corrected at the age of 14 months (Figure 1a). Her motor milestones were delayed as she sat independently at the age of 10 months and walked unaided at the age of 19 months. Her language development was also delayed as first words appeared after the age of 2 years.

Physical examination revealed a sociable child, with mild dysmorphic facial and body features such as microcephaly, small eyes with epicanthal folds, flat nasal bridge, moderate micrognathia and clinodactyly of 5^th^ finger. Her weight was 12 kg (10-25^th^ percentile), height 86 cm (3-10^th^ percentile**)** and head circumference 46 cm (< 2^nd^ percentile).

On developmental examination she showed good social and communication skills but with poor cognitive and verbal abilities. Her vocabulary consisted of around 20 simple words and two to three “2-word phrases”. According to Bayley Scales of Infant Development [4], her mental IQ score was 68.

Neurological examination revealed central generalized hypotonia without asymmetries or focal signs. Brain MRI scan showed mild dilation of the subarachnoid space. Audiological examination showed conductive hearing impairment and short external acoustic canal. Visual assessment revealed hypermetropic astigmatism. Biochemical tests, bone age, extensive metabolic investigation (including plasma aminogram, urine mucopolysaccharides, organic acids and lysosomal enzymes) and kidney-liver-spleen ultrasound proved normal.

She was re-evaluated at the age of 5 years and 5 months. She attended mainstream kindergarten and received speech therapy twice a week on regular basis. She remained sociable with mild microcephaly (HC = 48 cm, 3^rd^ percentile**)** and the same mild dysmorphic features (small eyes with marked epicanthus, moderate micrognathia, widely spaced nipples and clinodactyly of 5^th^ finger) (Figures 1b and c). Her speech was delayed consisting of small phrases with morphological and phonological disturbances. Her overall development was equivalent to a 4 year-old level with mental IQ score = 74. Her weight was 18 kg (25^th^ percentile) and her height 105 cm (3^rd^ percentile). Her hypermetropic astigmatism was corrected with glasses. Biochemical and endocrinological re-evaluation proved normal.

### Methods and results

Blood chromosome analysis was performed in the patient and her parents using high-resolution banding techniques. Twenty metaphases were analyzed from each subject by GTG- banding.

Molecular karyotyping was carried out on the DNA extracted from whole blood of the patient and both her parents according to standard procedures. All the experiments were conducted through oligonucleotide array-CGH platforms (SurePrint G3 Human CGH Microarray, 4x180K, Agilent Technologies, Santa Clara, CA). Briefly, 500 ng of proband DNA and of a sex–matched reference DNA (NA10851, Coriell Cell Repositories) were processed according to the manufacturer’s protocol. Fluorescence was scanned in a dual-laser scanner (DNA Microarray Scanner with Sure Scan High-Resolution Technology, Model G2565CA, Agilent Technologies, Santa Clara, CA) and the images were extracted and analyzed through Agilent Feature Extraction Software (v10.5.1.1). Graphical overview was obtained using the Genomic Workbench (v6.5) software. Changes in test DNA copy number at a specific locus were observed as the deviation of the log_2_ ratio value of 0 of at least three consecutive probes. The quality of each experiment was assessed by using a parameter provided by Agilent software (QC metric) and on the basis of DNA quality. Copy number changes identified in the samples were evaluated by using the UCSC Genome Browser website (http://genome.ucsc.edu) and the Database of Genomic Variants (http://projects.tcag.ca/variation). The positions of oligomers refer to the Human Genome February 2009 (versions NCBI 37, hg19) assembly. The DECIPHER (http://decipher.sanger.ac.uk/application) database was used to support genotype-phenotype correlation.

The array-CGH results were confirmed by copy number profiling using a qPCR method as previously described [5]. Two genes which map, the first at the beginning (GJA3 gap junction, alpha 3 = 20.712,394-20,735,188) and the second at the end (LINC00540 long intergenic non-protein coding RNA 540 = 22,681,609-22,850,660) of the putatively deleted region, were amplified with specific primers using the LightCycler® FastStart DNA MasterPLUS SYBR Green I mix (Roche Applied Science, Roche Diagnostics S.p.A., Monza, Italy). The real-time reactions were analyzed on a LightCycler® 1.5 (Roche Diagnostics GmbH, Mannheim, Germany). The concentration of the DNA samples was adjusted by including two reference genes, EIF3L and KDELR3, that are located on chromosome 22. Relative quantification, in respect to a calibration curve used to establish efficiency, was utilized to detect the number of copies of DNA targets per diploid genome. All PCR experiments were replicated three times. qPCR results demonstrated the presence of a single copy of the two target genes, per diploid genome of the fetus, confirming the array-CGH data (data not shown).

**Figure 2:**
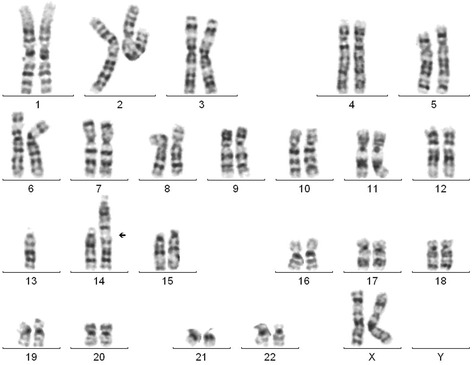
**A karyotype showing the unbalanced translocation between the two chromosomes 13 and 14.**

**Figure 3:**
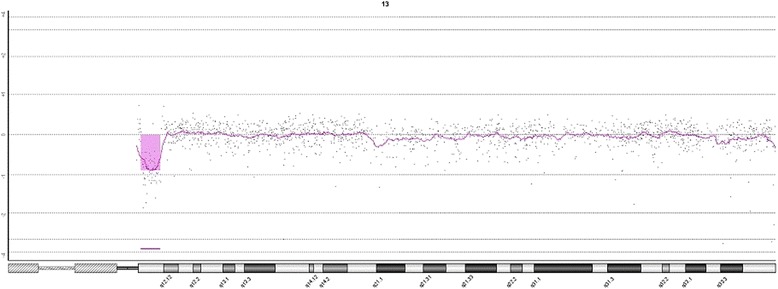
**Array-CGH profile of chromosome 13 showing a 2.9 Mb interstitial deletion.** At the top, the enlarged view of the rearrangement as provided by Agilent Technologies, CGH Analytics 3.5.14.

Cytogenetic analysis of the patient revealed a balanced translocation between acrocentric chromosomes 13 and 14. Chromosomal analysis of her parents showed normal karyotype and determined that the abnormality was *de novo*. The karyotype according to ISCN was 46,XX,der(13;14)(q12.11;qter)dn (Figure 2). Further genetic investigation was performed with the use of array-CGH. Molecular karyotyping revealed a deletion on the long arm of chromosome 13 as involving sub-bands 13q12.11, a deletion of about 2.9 Mb (chr13: 19938561–22840254) (Figure 3). A benign copy number variation (CNV) duplication in cytoband 15q11.2 (chr15:20602842–22509395) was also observed. This CNV contains 1.9 Mb of genomic material and includes genes BCL8 and POTEB. In the Genomic Data of Variants (http://projects.tcag.ca/variation/), this kind of CNV is described as a genetic polymorphism often seen in healthy individuals, and therefore is not expected to be linked to any pathologic phenotype.

## Discussion

Proximal deletions in the 13q12.11 region are very rare. Much larger deletions including this region have been described and are associated with complex phenotypes of mental retardation, developmental delay and various others anomalies.

We describe a 5 ½ years old girl presenting with developmental delay, microcephaly, small eyes with marked epicanthus, flat nasal bridge, moderate micrognathia, strabismus, broad nasal bridge, widely spaced nipples, posteriorly rotated ears, and clinodactyly of the 5^th^ finger.

To date, two other cases with interstitial deletion within the 13q12.11 region have been reported and presented similar developmental and facial characteristics (Table 1) [2,3]. Kaloustian et al. [2] reported a patient with mild developmental delay, craniofacial dysmorphism, pectus excavatum, narrow shoulders, malformed toes, and café-au-lait spots. Array- CGH analysis revealed a de novo deletion spanning 2.1 Mb within cytogenetic band 13q12.11. The deleted region encompassed 16 genes. The second case, a patient with a 2.9 Mb deletion, was reported by Tanteles et al. [3]. It was a 16-year-old boy with a degree of facial dysmorphism, scaphocephaly, torticollis and near normal development and intellect. The deletion produced hemizygoxity for 19 known genes.

**Table 1:** **Phenotype of the three patients with deletion of the 13q12.11 region**

	**Tanteles et al.**	**Der Kaloustian et al.**	**Our patient**
Size of deletion, Mb	2.9	2.1	2.9
Inheritance	De novo	De novo	De novo
Age at diagnosis	16 years	3 years and 2 months	3 years and 6 months
Sex	M	M	F
Birthweight, g	2500	2950	3150
Mental retardation	-	-	+
Speech delay	+	+	+
Hypotonia	-	+	+
Failure to thrive	-	-	+
Short stature	-	-	-
Brain anomalies	-	-	Dilatation of subarachnoid space and temporal section of both lateral ventricles
Cardiac anomalies	Murmur, abnormal aortic valve	-	-
Ophthalmic abnormalities	Divergent squint, hypermetropia	-	Divergent squint, high hypermetropic astigmatism
Oropharyngeal dyspasia	-	-	Incomplete cleft palate, short external acoustic canal
Scoliosis	+	-	-
Renal anomalies	-	-	Small kidney cysts
Clinodactyly	+	-	Fifth finger
Feet anomalies	Calcaneovalgus deformity of the left foot	-	-
Microcephaly	-	-	+

**Figure 4:**
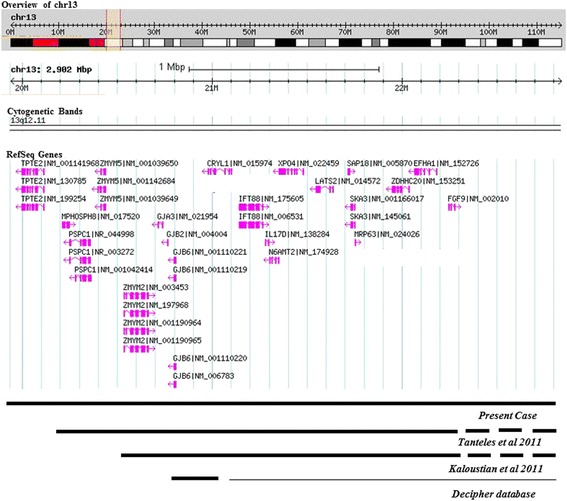
**Schematic representation of our patient’s deletion showing 2.9 Mb from chromosome 13 (chr13), positions 19,938,561-22,840,254 according to Database of Genomic Variants genome browser image Build GRCh37: Feb. 2009, hg 19.** The deleted area of the two other patients and the genes involved are also noted.

According to the DECIPHER database six cases are mentioned in the area of our interest, within the area of 20,797,139 to 21,099,089, (cases: 1032, 261403, 273408, 285395, 288952, 289764) with micro deletions ranging from 180 kb to 290 kb, presenting major figures such us intellectual disability, delayed speech and language development and according to the NCBI RNA Reference Sequences Collection (RefSeq), the deleted area of our patient encompasses 20 transcribed genes and (Figure 4). Four of these genes *GJA3*, *GJB2*, *GJB6*, and *FGF9* are known to be associated with monogenic disorders and have potential clinical significance. *GJA3* causes autosomal dominant zonular pulverulent cataract-3 [6] while *GJB2* and *GJB6* genes cause autosomal recessive sensorineural hearing loss worldwide [7–9]. In addition, autosomal dominant mutations in *GJB2* are responsible for Keratitis-Ichthyosis-Deafness syndrome and rare forms of palmoplantar keratoderma associated with sensorineural hearing loss [10–12]. Mutations in *GJB6* may also cause a form of hidrotic ectodermal dysplasia known as Clouston syndrome [13]. Our patient did not show signs of sensorineural hearing loss nor Clouston syndrome. A mechanism for an autosomal recessive disorder could be a large deletion on the one chromosome and a point mutation on the other chromosome.

Other known transcribed genes in the deleted region are the *FGF9*, *LATS2* and *IFT88 genes. FGF9* is associated with one form of multiple synostoses syndrome [14] while loss of heterozygosity at the *LATS2* gene is associated with non-small cell lung cancer [15]. *IFT88* is considered a candidate gene for renal disease [16]. These three disorders are inherited in an autosomal dominant fashion, but it is possible that haploinsufficiency due to deletion on the one chromosome is not a causative mechanism for these particular disorders.

## Conclusion

Comparison of the extent of the reported deletions and the phenotype of the patients indicates that our patient with a 2.9 Mb 13q12.11 deletion does not represent a shortest region of overlap (SRO) of a microdeletion syndrome. The deletion appears, however, associated with a number of abnormalities such as mild developmental delay, speech problems, microcephaly, and mild dysmorphic features. The description of more patients with deletions of the 13q12.11 region will allow a more precise genotype-phenotype correlation.

## Consent

Written informed consent was obtained from the parents of this patient for publication of this case report. A copy of the written consent is available for review by the Editor-in-Chief of this journal.

## Abbreviations

array-CGH: Array based comparative genomic hybridization
qPCR: Quantitative polymerase chain reaction
CNV: Copy number variation
ISCN: International System for Human Cytogenetic Nomenclature
SRO: Shortest region of overlap
